# Anaesthesia and cancer: can anaesthetic drugs modify gene expression?

**DOI:** 10.3332/ecancer.2020.1080

**Published:** 2020-07-27

**Authors:** Aida Raigon Ponferrada, Jose Luis Guerrero Orriach, Alfredo Malo Manso, Enrique Sepúlveda Haro, Salvador Romero Molina, Ana Fontaneda Heredia, Manolo Baena Lopez, Jose Cruz Mañas

**Affiliations:** 1 Institute of Biomedical Research in Malaga [IBIMA], Malaga 29010, Spain; 2 Department of Anaesthesiology, Virgen de la Victoria University Hospital, Malaga 29010, Spain; 3 Department of Pharmacology and Pediatrics, School of Medicine, University of Malaga, Malaga 29010, Spain; 4 Member of COST Action 15204; 5 Department of Urology. Virgen de la Victoria University Hospital, Malaga 29010, Spain; 6 Department of Anaesthesiology, Complejo Hospitalario de Jaen, 29010, Spain; 7 Department of Oncology, Virgen de la Victoria University Hospital, Malaga 29010, Spain

**Keywords:** anaesthesia, analgesia, cancer recurrence, metastases, anaesthetics, regional anaesthesia, opioid, NSAIDs

## Abstract

Cancer remains a primary cause of morbidity and mortality worldwide, and its incidence continues to increase. The most common cause of death in cancer patients is tumour recurrence. Surgery is the gold standard in the treatment of most tumours. However, cancer surgery can lead to the release of tumour cells into the systemic circulation. Surgical stress and several perioperative factors have been suggested to boost tumour growth, thereby increasing the risk of metastatic recurrence.

Preclinical and clinical studies suggest that anaesthetics and adjuvants administered during the perioperative period may impact cancer recurrence and survival. This document summarises the current evidence regarding the effects of anaesthetic drugs and analgesic techniques on the immune system, systemic inflammatory response and tumour cells, as well as their impact on cancer recurrence.

## Introduction

Cancer is the second cause of mortality in developed countries, with most deaths caused by metastasis [1]. Surgery is one of the factors involved in metastatic spread since it may facilitate the release of cancer cells into the bloodstream during tumour manipulation. Another factor related to cancer spread is the systemic inflammatory response associated with perioperative surgical stress. Consequently, surgery implies two factors related to metastatic progression, i.e., the presence of cancer cells with metastatic potential and an appropriate microenvironment for their growth. Among that can affect metastatic progression of tumour cells is the anaesthetic technique [2].

The anaesthetic technique can influence patients’ neuroendocrine and immune responses during surgery. Surgical stress can suppress the antitumour immune response and stimulate the hypothalamic-pituitary-adrenal (HPA) axis, which, along with the activation of the sympathetic nervous system (SNS), also regulates the immune response. The activation of the HPA and the SNS leads to the suppression of cell immunity (CI) as well as the release of catecholamines and prostaglandin E2 [3]. In turn, these molecules increase immunosuppressant cytokines such as interleukin (IL): IL-4 and IL-10, TGF-β (transforming growth factor-beta) and vascular endothelial growth factor (VEGF); as well as pro-inflammatory cytokines, such as IL-6 and IL-10, which promote tumour angiogenesis and facilitate the development of metastases [4].

Anaesthetic agents vary in their ability to induce immunomodulation and boosting of tumour growth factors. Pre- and postoperative opioids are able to inhibit the humoral immune response and can have pro-angiogenic effects, which promote tumour cell growth [5, 6]. On the other hand, regional anaesthesia preserves CI and reduces the surgery-induced neuroendocrine response by debilitating afferent activations mediated by the HPA axis and SNS neural responses. Regional anaesthetic techniques have been associated with lower rates of cancer recurrence [7, 8].

Despite the fact that some studies have shown significant benefit in terms of cancer recurrence a recent metaanalysis shows that RA has no overall survival, recurrence- free survival or biochemical recurrence-free survival benefit [9].

In this review, we aim to provide more information regarding the role of surgery and anaesthesia in different aspects of tumour recurrence.

## Surgical stress

Surgery remains the primary treatment for most cancer patients. However, the surgical stress leads to immune suppression, allows tumour cell adhesion and increases the release of metalloproteases (MMP) and VEGF. These factors increase the mobility and invasiveness of cancer cells and neovascularisation, and promote cancer progression and metastasis formation.

The first line of defence against the development of primary tumours and metastases are natural killer (NK) cells [10]. The changes in the activity of NK cells depend on both the intensity of the surgery and the magnitude of the stress response, which increases the release of catecholamines and prostaglandins by activating the HPA axis and SNS [11]. From a clinical perspective, surgery leads to decreased circulation of NK and T cells via the induction of apoptosis. Surgical stress increases the amount of Th2 lymphocytes and decreases the amount of Th1 lymphocytes, thereby reducing the Th1/Th2 ratio, which eventually leads to CI suppression [12]. Moreover, the levels of immunomodulatory cytokines such as IL-2, IL-12 and interferon-γ (IFN-γ) decrease, whereas anti-inflammatory cytokines such as IL-10 increase [13].

## Oxidative stress, inflammation and molecular regulators

Oxidative stress and inflammatory response are among the most important factors that influence the development, growth and metastatic spread of malignant tumours. Oxidative stress can be induced in tumour cells by overproduction of reactive oxygen species (ROS) due to downregulation of NADPH oxidase [14]. It can also be induced by overexpression of thymidine phosphorylase, as observed in most carcinomas [15].

In general, tumours quickly outgrow their blood supply, leading to glucose deprivation and hypoxia. The lack of glucose induces oxidative stress and depletes intracellular pyruvate in cancer cells, thus preventing the breakdown of endogenous oxygen radicals [16]. Oxygen radicals damage DNA, causing filament tears, guanine and thymine alterations and exchanges of sister chromatids. The genetic instability secondary to oxidative stress increases the malignant potential of tumours. Furthermore, oxidative stress can activate several transcription factors, including NF-κB, AP-1, p53 and hypoxia-inducible factor-1 α (HIF-1) [17].

In tumour cells, the expression of HIF promotes cell proliferation and induces the secretion of angiogenic factors, including VEGF and angiopoietin-2. Therefore, hypoxia is strongly associated with tumour progression and metastatic spread [18]. A comprehensive review by Tavare et al [19] described the direct effects of anaesthetics on HIF-1, which is upregulated by inhalational anaesthetics, and inhibited by propofol. Oxidative stress and inflammation are interrelated. Oxidative stress activates inflammatory pathways that transform normal cells into tumour cells, increasing their chances of survival, proliferation, chemo- and radio-resistance, invasiveness and angiogenesis, as compared to stem cells [20].

## Effects of anaesthetic agents on immune function and tumour development

### Halogenated anaesthetics

Several studies showed that halogenated anaesthetics inhibit the activity of the immune system. Their impact on the activity of NK and T cells is time- and dose-dependent [21]. Volatile anaesthetics also inhibit various lymphocyte functions, including proliferation and cytokine production [22].

Sevoflurane has been demonstrated to increase the levels of pro-tumourigenic cytokines and MMP [23]. Isoflurane reduces the activity of NK cells, induces T cell and B cell apoptosis and reduces the Th1 /Th2 ratio [24].

Unlike total intravenous anaesthesia (TIVA), exposure to sevoflurane increases the levels of proteins such as cytoplasmic HIF-2α and nuclear p38, which are both associated with a worse prognosis in cancer patients [25]. Halothane reduces the activity of NK cells and increases HIF-1α expression [26]. Conversely, propofol prevents the activation of HIF-1α induced by isoflurane, which is related to a partial reduction of the malignant behaviour of cancer cells [27]. Isoflurane is associated with higher levels of HIF-1α and increased proliferation and migration of cancer cells. Sevoflurane induces T cell apoptosis and increases the rate of expression of HIF-1α [28].

Isoflurane increases the malignant potential of cancer cells through the upregulation of insulin-like growth factor (IGF)-1 and its receptor (IGF-1R), as well as VEGF, angiopoietin-1, MMP-2 and MMP-9. Moreover, isoflurane exposure leads to resistance to apoptosis through a caveoline-1-dependent process [23, 29].

Nitrous oxide (N2O) disrupts the synthesis of DNA, purines and thymidylate, which can induce oncogenesis [30]. As demonstrated in an in vivo model, N2O suppresses chemotaxis, which is potentially the strongest stimulator for the development of liver and lung metastases after surgery [31].

### Intravenous anaesthetics: propofol, ketamine and thiopental

Intravenous hypnotics have multiple effects on the immune system. Unlike propofol, ketamine and thiopental suppress the activity of NK cells [32]. Ketamine induces apoptosis in lymphocytes via a mitochondrial pathway and inhibits the functional maturation of dendritic cells, while thiopental protects T cells from apoptosis via the induction of thermic shock proteins [33].

On the one hand, ketamine reduces the synthesis of pro-inflammatory cytokines, such as IL-6, and tumour necrosis factor α (TNF-α). On the other hand, thiopental inhibits the function of neutrophils and suppresses the activation of nuclear factor kappa B (NF-κB). This factor is associated with inhibition of the activity of the NF-κB reporter gene, which leads to activation of T cells, secretion of IL-2, IL-6 and IL-8, as well as overexpression of IFN-γ [34].

Apparently, propofol displays a different profile since its protective effects are exerted by other mechanisms, including anti-inflammatory effects, COX-2 inhibition and PGE-2 reduction, increased cytotoxic T lymphocyte activity, and decreased pro-inflammatory cytokines [35]. Propofol does not affect the Th1/Th2 ratio [36], and is weakly bound to β-adrenoreceptor, producing a β-blocking effect that improves anti-tumour immunity and preserves the function of NK cells [37]. Patients receiving perioperative β-blockers have a lower recurrence of metastases after surgery [38].

Propofol conjugates (propofol-docosahexanoate and propofol-eicosapentanoate) have been shown to inhibit cell adhesion and migration, and to induce apoptosis in cancer cells [39]. Propofol reduces cytokine concentrations (IL-1, TNF-α and IL-6), and stimulates the secretion of nitrous oxide in neutrophils [40]. Propofol concentrations of 1–5 mg/mL were found to decrease the invasiveness of cancer cells. Moreover, continuous infusion of propofol can inhibit the development of lung metastases. Wigmore *et al* [41] retrospectively compared the long-term survival of patients under general anaesthesia using halogenates versus TIVA in cancer surgery. They concluded that the modality of anaesthesia was significantly associated with patient survival, which was higher in the TIVA group. Working under this assumption, Enlund *et al* [42] observed that 1-year survival was almost 10% higher in cancer surgeries where propofol was used as an anaesthetic.

In contrast with these findings, a recent retrospective study in lung cancer patients found no outcome improvements when comparing TIVA versus inhalation anaesthesia [43].

### Opioids

Opioid analgesics can affect tumour development via the modulation of cell proliferation and apoptosis. Morphine suppresses the activity of NK cells and the differentiation of T cells, promotes lymphocyte apoptosis and decreases toll-like receptor 4 (TLR4), expressed in macrophage membranes [44]. Likewise, fentanyl and sufentanil decrease the activity of NK cells but increase the number of regulatory T cells. Morphine stimulates the proliferation and angiogenesis of endothelial cells by activating mitogen-activated protein kinase/extracellular signal-regulated kinase phosphorylation via Gi/Go-coupled G protein receptors and nitric oxide in these microvascular endothelial cells [45, 46]. The promotion of tumour growth is mediated by AKT; extracellular signalling is mediated by ERK (extracellular signal-regulated kinase); the promoting effects of cell apoptosis are mediated by the inhibition of NF-κB, increase in Fas expression, p53 stabilisation, p38 activation and c-jun-N kinase (JNK) [47]. These effects include apoptosis inhibition via AKT activation, and promotion of cell cycle progression via the increase of cyclin D1 [48].

Studies suggest that sufentanil also inhibits leukocyte migration [49], alfentanil decreases the activity of NK cells and remifentanil was reported to suppress the activity of NK cells and lymphocytic proliferation in an in vitro model [50]. In addition, fentanyl showed anti-tumour effects in colorectal cancer cells in vitro. Its use is associated with reduced tumour cell migration and invasion via the inhibition of downregulation of E-26 transformation-specific sequence-1 into activated serine/threonine-kinase protein kinase B-raf (BRAF) – lncRNA [51].

Opioid-induced cell proliferation is likely to be concentration- and time-dependent. When low concentrations or a single dose of opioids are used, tumour growth is stimulated. Conversely, high concentrations or chronic opioid exposure leads to tumour growth inhibition [52]. In addition, morphine proved to inhibit the expression and secretion of MMP-2 and MMP-9 in breast cancer cells in a time- and dose-dependent manner.

Another study showed that fentanyl inhibits tumour growth and cell invasion in colorectal cancer due to downregulation of miR-182 and MMP-9 expression by β-catenin. A recent study demonstrated that sufentanil does not affect the rate of apoptosis or the cell cycle distribution in colon and pancreatic cancer cells in vitro when clinical concentrations were used. MMP activity cannot be reverted by naloxone, which indicates that the inhibition of MMP secretion by morphine is not mediated by opioid receptors [53].

Furthermore, overexpression of μ-opioid receptor (MOR) promotes tumour growth and metastasis in several cancer cell types [54]. The activation of AKT and mTOR is associated with cell proliferation and migration which, in turn, are related to MOR overexpression [55].

Treatment with methyl-naltrexone (MNTX), an opioid antagonist, inhibits tumour cell invasion and implantation, while continuous infusion of MNTX decreases primary tumour growth and development of lung metastasis. Clinically, MNTX has been found to be associated with higher overall survival rates in patients with advanced cancer, which supports the hypothesis that MOR is involved in tumour progression [56]. Treatment with morphine, both prior to and after surgery, significantly reduced stress-induced corticosteroids in rats [57]. This finding suggests that the preoperative administration of morphine may play a key role in protection against surgery-induced metastasis [58].

### NSAIDs and COX-2 inhibitors

The induction of COX-2, which is frequently observed in cancer, has a role in immune resistance. COX-2 inhibitors increase the cytotoxicity of NK cells [59]. Moreover, when combined with β-adrenergic antagonists, they proved to decrease metastases in animal models [18]. A selective COX-2 inhibitor can suppress the release of prostaglandin E2 (PGE2) and promote immune responses against cancer cells [60].

The use of non-steroid anti-inflammatory drugs (NSAIDs) in preoperative medication increases cell immunity in cancer tissues [61]. PGE2 is a tumour-derived angiogenic factor independent of VEGF. PGE2 synthesis is controlled by COX-2 expression, and COX-2 inhibition blocks VEGF, leading to angiogenesis inhibition, tumour growth and metastasis formation [62].

Recently, study indicated that morphine enhanced TNBC metastasis and angiogenesis while ketorolac suppressed this effect. Mechanistically, this may be related to the enhancement of TSP-1 synthesis after ketorolac administration which further de-activated PI3K/AKT/c-Myc pathway [63].

However, in a recent clinical trial reported that a single administration of 30 mg of ketorolac tromethamine before surgery does not increase disease-free survival in high-risk breast cancer patients. Overall survival difference between ketorolac tromethamine group and placebo group was not statistically significant [64]

### Local anaesthetics

Local anaesthetics block voltage-dependent sodium channels and may inhibit tumour growth. Lidocaine, ropivacaine and bupivacaine inhibit cell proliferation and differentiation, are cytotoxic for mesenchymal stem cells in vitro, and play a key role in tumour growth and metastasis development in cancer cells [65].

Locally-administered lidocaine inhibits epidermal growth factor receptor, which is a target molecule of many cancer drugs. A study that assessed the direct effect of local anaesthetics showed that lidocaine and bupivacaine induce apoptosis in cancer cells both in vivo and in vitro, suggesting potential benefits for cancer surgery [66]. Lidocaine and tetracaine, which inhibit motor proteins of kinesin, reduce the formation and activity of tubulin microtentacles. Therefore, these drugs may have a new ability to decrease metastatic spread in cancer [67]. In addition, lidocaine produces DNA demethylation in breast cancer cells in vitro [68]. Moreover, lidocaine, ropivacaine and bupivacaine reduce cell proliferation at concentrations of 100 μM, leading to cell cycle delay or arrest at G0/S-1 phases [69].

A recent study demonstrated that topic lidocaine increases the activity of NK cells against cancer in vitro by releasing lytic granules [70]. Furthermore, results from basic science studies reveal a promising role of local anaesthetics regarding the reduction of tumour recurrence [71].

## Anaesthetics and genetic implications

The possibility that many of the beneficial or harmful effects related to the drugs used in the anaesthetic procedure, have a genetic and molecular basis is increasingly accepted [72]. The oncological disease could be mediated by specific genes or molecular pathways.

The relationship between genes and protein coding is widely known (DNA -> mRNA -> protein). Variations in the signalling pathways and the cells genetic material made by cancer is one of the objectives of study and development of current treatment.

Nevertheless, less than 2% of the mammalian genome encodes proteins, which means that >90% represents non-coding RNA (ncRNA) [73]. ncRNAs are involved in the control of development, differentiation, metabolism, cell growth and tumour progression [74]. In general, ncRNAs are classified into two groups based on their length: small ncRNAs (sncRNAs) and long ncRNAs (lncRNAs). sncRNAs include microRNA (miRNA), transfer RNA (tRNA) and some ribosomal RNA transcripts. Interplay patterns between lncRNAs and miRNAs appear to be crucial events in cancer progression.

Emerging data support the involvement of lncRNA in tumour-stroma communication, a potentially important landmark in cancer progression. Recently, Sang *et al* [75] demonstrated that lncRNA participates in the activation of calcium-dependent kinase (CamK-A), which is highly activated in several cancers and involved in remodelling the tumour microenvironment through the activation of calcium (Ca2+), thereby promoting macrophage recruitment, angiogenesis and cancer progression.

Different miRNAs have been identified in biological fluids, such as urine and blood [76], serving as potential biomarkers in the diagnosis and prognosis of cancer. More recently, lncRNAs have been highlighted as potential biomarkers and cancer targets in precision medicine due to their specific expression patterns in tumour cells [77]. The role of LncRNAs in cancer disease has been known as different studies have shown its role as a diagnostic and prognostic element related to resistance to several drugs, through the modulation in the expression of drug transporters, and have been identified in signalling pathways that contribute to oncogenic survival, cell cycle, and apoptosis. In this line, lncRNA-specific therapeutic approaches target lncRNA-mediated functions and pathways through gene silencing and structure disruption mechanisms[78].

The genetic modifications in LncRNAs, trying to modulate a group of genes and acting on their functions, are the basis of future therapeutic objectives that could be reached.

In the regulation of gene expression by circular RNAs (circRNAs) through disease-related miRNAs, a complex network of miRNA-circRNA-ncRNA interacting at a protein level is formed, affecting a wide range of human diseases, particularly cancer. Likewise, circRNAs also have oncogenic and proto-oncogenic roles. Fusion circRNAs (f-circRNAs) result from cancer-associated genomic translocations and have tumour-promoting properties, including an increase in cell viability, a higher resistance to cancer therapy, and in vivo cell transformation into leukaemia cells [79]. circRNA expression has been demonstrated in different types of human cells [80], playing important physiological roles by regulating cell proliferation and haematopoiesis [81]. In addition, circRNAs have been implicated in the mediation of immune responses. For instance, circRasGEF1B upregulates the expression of TLR4/LPS-induced ICAM-1 mature mRNA stability, thus controlling the innate immune responses. Moreover, cellular mechanism allows to recognise foreign circRNAs through intron identity, which enables the identification of self and foreign circRNAs within the cytoplasm of the host [82].

Retrospective analyses suggest that an anaesthetic-analgesic technique during cancer surgery may affect recurrence/metastasis. This could involve direct effects on cancer cells. An interesting research objective is the modification of gene expression as a target of anaesthetic-analgesic drugs.

On the other hand, propofol has a potential antitumour effect, mainly due to the regulation of miRNA expression and transference. Accordingly, Wang *et al* [83] found that propofol suppresses cell proliferation and invasion in pancreatic cancer cells through the upregulation of miRNA-133a expression.

Furthermore, Xu *et al* [84] found that propofol induces the upregulation of miRNA let-7i expression and cell apoptosis in epithelial ovarian cancer cells in vitro.

Propofol increases miRNA-218 and miRNA-451 expression, whereas it reduces MMP-2 protein expression and cancer cell proliferation in vitro [85]. Likewise, Zhang *et al* [86] found that propofol reduces the invasiveness of hepatocellular carcinoma cells, partly due to the downregulation of MMP-9 by miRNA-199a.

In addition, the low expression of miRNA contributes to the antitumour effect of propofol. Accordingly, miRNA-21 is overexpressed in the early stages of pancreatic cancer [87]. Propofol inhibits miRNA-21 and suppresses the invasion of pancreatic cancer cells. In fact, it is thought that propofol inhibits the expression of miRNA-21 and reduces the expression of Slug, resulting in an increase in Slug-dependent PUMA (p53 proapoptotic target gene) and in the expression of E-cadherin [88]. The activation of PUMA and E-cadherin is involved in the inhibition of cell apoptosis. Therefore, propofol induces apoptosis and inhibits the invasion of pancreatic cancer cells through miR-21/Slug/E-cadherin and miR-21/Slug/PUMA signalling pathways [87].

Anaesthetic drugs may affect oncogene overexpression in certain types of cancer. Propofol can inhibits the expression of the androgen receptor in vitro, thus indicating a potential positive effect, as androgenic stimulation is involved in prostate cancer progression [16]. Furthermore, propofol has been shown to reduce of HIF-1a levels in cancer cells in vitro, with potential inhibitory effects on angiogenesis and, therefore, on tumour growth [89]. Volatile anaesthetics are known to be protective against ischaemia-reperfusion injury in a variety of clinical contexts and organ systems. This protection is associated with an induced expression of the angiogenesis-regulating factor HIF-1a, a mechanism that may be protective in the setting of reperfusion injury, but which promotes malignant recurrence in cancer surgery. Isoflurane has been found to increase the expression of HIF in prostate and renal cell carcinoma cells in different studies, and this has been associated with increased cancer cell migration and proliferation.

Opioids are related to modulation in cancer progression. Morphine, even when it was given in a low dose, can change the expression of gene groups, and induce metastasis (in vitro) [90]. The NET1 gene, belongs to this genetic group, overexpressed in breast and gastric adenocarcinoma cells through the Serial Analysis of Gene Expression database. The NET1 gene has a key role in the organisation of the actin cytoskeleton and, thus, in the ability of cancer cells to migrate and invade tissues, and even more, can help identify breast cancer patients at high risk of metastasis [91].

There is evidence supporting that a single dose of morphine can alter the expression of two major gene groups: regulators of proteins involved in mitochondrial pathways, and cytoskeleton-related proteins [92,93]. However, it has not been determined whether it modifies any cell function.

TNF-α increases the expression of intracellular adhesion molecule-1 (ICAM-1), a receptor required for leukocyte adhesion and tumour invasion. TNF-α also activates Src protein tyrosine kinase, which is a regulator of endothelial permeability. Src is involved in extravasation of cancerous cells, which is necessary for solid tumour metastasis [94]. Src is also involved with regulation of the cytoskeletal changes required for cell migration by phosphorylation of the proteins involved in focal adhesions and actin binding [95]. Lidocaine and ropivacaine have been reported to decrease cancer cell migration by inhibiting Src activation induced by tumour necrosis factor-α and phosphorylation of intercellular adhesion molecule-1 [96]. A later study showed that these local anaesthetics may have an anti-inflammatory effect, since in endothelial cells they effectively block inflammatory signaling of TNFα by attenuating the recruitment of p85 in the TNF-1 receptor. The resulting decrease in Akt, endothelial nitric oxide synthase, and Src phosphorylation reduced neutrophil adhesion and endothelial hyperpermeability [97].

## Conclusion

There is growing evidence that the anaesthetic technique and anaesthetics may play a relevant role in tumour dissemination and relapse in the long term. The perioperative use of anaesthetic/analgesic techniques with protective effects on antimetastatic immune response may reduce tumour progression. To understand the mechanisms is important in order to study the genetic implications regarding anaesthetics drugs. New studies are necessary, the effects of anaesthetic drugs on the genetics of cancer need to be defined. The results of these studies may provide an answer if the data in animal models and in vitro studies can be applied in clinical practice and these studies may provide a new therapeutic target in cancer cells.

## Sources of funding

This paper has been supported by the Anaesthesia Department and the Institution for Clinical Science. No external funding has been received.

## Competing interests

The authors declare that they have no competing interests.
